# Genetic mechanisms underlying yield potential in the rice high-yielding cultivar Takanari, based on reciprocal chromosome segment substitution lines

**DOI:** 10.1186/s12870-014-0295-2

**Published:** 2014-11-18

**Authors:** Toshiyuki Takai, Takashi Ikka, Katsuhiko Kondo, Yasunori Nonoue, Nozomi Ono, Yumiko Arai-Sanoh, Satoshi Yoshinaga, Hiroshi Nakano, Masahiro Yano, Motohiko Kondo, Toshio Yamamoto

**Affiliations:** NARO Institute of Crop Science, Tsukuba, Ibaraki 305-8518 Japan; National Institute of Agrobiological Sciences, Tsukuba, Ibaraki 305-8602 Japan; Institute of the Society for Techno-innovation of Agriculture, Forestry and Fisheries, Tsukuba, Ibaraki 305-0854 Japan

**Keywords:** Chromosome segment substitution lines (CSSLs), Quantitative trait locus (QTL), Rice, Yield potential

## Abstract

**Background:**

Increasing rice yield potential is a major objective in rice breeding programs, given the need for meeting the demands of population growth, especially in Asia. Genetic analysis using genomic information and high-yielding cultivars can facilitate understanding of the genetic mechanisms underlying rice yield potential. Chromosome segment substitution lines (CSSLs) are a powerful tool for the detection and precise mapping of quantitative trait loci (QTLs) that have both large and small effects. In addition, reciprocal CSSLs developed in both parental cultivar backgrounds may be appropriate for evaluating gene activity, as a single factor or in epistatic interactions.

**Results:**

We developed reciprocal CSSLs derived from a cross between Takanari (one of the most productive *indica* cultivars) and a leading *japonica* cultivar, Koshihikari; both the cultivars were developed in Japan. Forty-one CSSLs covered most of the Takanari genome in the Koshihikari background and 39 CSSLs covered the Koshihikari genome in the Takanari background. Using the reciprocal CSSLs, we conducted yield trials under canopy conditions in paddy fields. While no CSSLs significantly exceeded the recurrent parent cultivar in yield, genetic analysis detected 48 and 47 QTLs for yield and its components in the Koshihikari and Takanari backgrounds, respectively. A number of QTLs showed a trade-off, in which the allele with increased sink-size traits (spikelet number per panicle or per square meter) was associated with decreased ripening percentage or 1000-grain weight. These results indicate that increased sink size is not sufficient to increase rice yield in both backgrounds. In addition, most QTLs were detected in either one of the two genetic backgrounds, suggesting that these loci may be under epistatic control with other gene(s).

**Conclusions:**

We demonstrated that the reciprocal CSSLs are a useful tool for understanding the genetic mechanisms underlying yield potential in the high-yielding rice cultivar Takanari. Our results suggest that sink-size QTLs in combination with QTLs for source strength or translocation capacity, as well as careful attention to epistatic interactions, are necessary for increasing rice yield. Thus, our findings provide a foundation for developing rice cultivars with higher yield potential in future breeding programs.

**Electronic supplementary material:**

The online version of this article (doi:10.1186/s12870-014-0295-2) contains supplementary material, which is available to authorized users.

## Background

Increasing crop productivity is a global challenge and is necessary for keeping pace with population growth worldwide [1]. More than half of the world’s population is in Asia, where rice is grown and consumed as a staple food [2]. The predicted population growth in Asia will require a 60–70% increase in rice production by 2050, but there is insufficient space for a corresponding increase in agriculture [3]. To meet the anticipated demand, it is necessary to increase rice production by improving potential rice yield per unit land area.

In the tropics, the yield potential of current high-yielding inbred rice cultivars is 10 t · ha^−1^ as unhulled rice under favorable irrigated conditions [4]. This yield potential was first attained by IR8, the first modern high-yielding cultivar released by the International Rice Research Institute (IRRI) in the late 1960s. The release of IR8 and subsequent high-yielding cultivars helped to more than double rice production over the past half century. This successful increase in production was called the “Green Revolution” in rice [5]. However, recent trends in yield in tropical environments indicate that yield potential has stagnated since the release of IR8 [6].

In temperate Japan, high-yielding rice has been developed using the *indica* and *japonica* cultivars since the 1980s [7]. The latest yield trials, conducted using recently developed high-yielding cultivars, produced nearly 10 t · ha^−1^ as brown rice (>12 t · ha^−1^ as unhulled rice yield) in eastern Japan [8] and >10 t · ha^−1^ as brown rice in western Japan [9]. Among the individual trials, a brown rice yield of 11.7 t · ha^−1^ was reported in western Japan [9]. To our knowledge, this represents the highest yield recorded in Japan to date, and was attained using Takanari, a high-yielding *indica* cultivar. Takanari is a semidwarf cultivar descended from high-yielding cultivars including IR8 [10]. Ecophysiological studies have characterized Takanari as having large sink size as a result of high spikelet number per panicle, strong source characteristics (e.g., high photosynthesis rate), and high carbohydrate translocation capacity [11-14]. Therefore, it is important to understand the genetic mechanisms underlying the high yield potential in Takanari to further improve this potential.

Over the past two decades, advances in molecular genetics technology using the complete rice genome sequence have facilitated genetic analyses, including the mapping and cloning of quantitative trait loci (QTLs) that control complex traits [15,16]. Chromosome segment substitution lines (CSSLs), which carry a specific donor chromosome segment in the genetic background of a recurrent cultivar, are powerful tools for enhancing the potential of genetic analysis. CSSLs are appropriate for detecting QTLs with both large and small effects that are often masked by other QTLs with large effects in primary populations, such as F_2_ populations and recombinant inbred lines [17,18]. Because yield is a highly complex trait that is controlled by a large number of QTLs with small effects, CSSLs are useful for understanding the genetic mechanisms underlying this characteristic. To date, several CSSLs have been developed in rice for several cross combinations [17,19-23], including reciprocal CSSLs [20,21]. Reciprocal CSSLs have the advantage of enabling evaluation of differences in allelic effects of QTLs in both genetic backgrounds. However, to our knowledge, genetic analysis of rice yield potential has not been conducted using reciprocal CSSLs. Therefore, the development of reciprocal CSSLs for yield trials using Takanari represents a promising approach.

In this study, we developed reciprocal CSSLs from a cross between Takanari and Koshihikari, a leading *japonica* cultivar, by repeated backcrossing, self-pollinating, and marker-assisted selection (MAS). The CSSLs in the Koshihikari background consisted of 41 lines covering the entire Takanari genome, and these are promising materials for detecting QTLs underlying high yield potential in Takanari. The CSSLs in the Takanari background consisted of 39 lines covering the entire Koshihikari genome, and they may enable detection of QTLs for increasing yield potential in Takanari. Yield trials using the reciprocal CSSLs revealed a number of QTLs associated with yield and its components in both genetic backgrounds. Our findings provide a foundation for developing rice cultivars with higher yield potential in future breeding programs.

## Methods

### Development of the CSSLs

**Figure 1:**
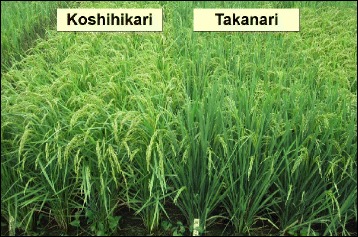
**Image of Koshihikari and Takanari plants.**

**Figure 2:**
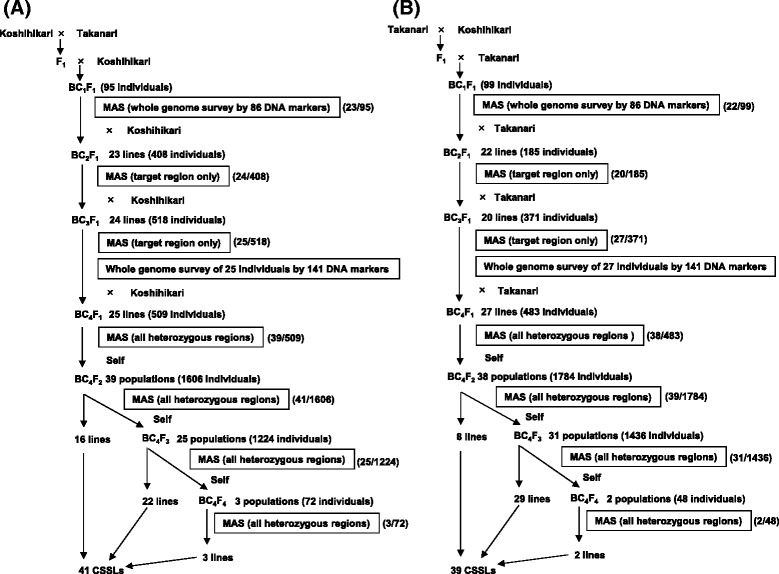
**Schematic of the development of the reciprocal chromosome segment substitution lines (CSSLs) between Koshihikari and Takanari.** CSSLs carrying a Takanari chromosomal segment in the Koshihikari genetic background **(A)** and a Koshihikari chromosomal segment in the Takanari genetic background **(B)**. The numerator and denominator in parentheses indicate the number of plants selected and the number investigated by marker-assisted selection (MAS), respectively. A total of 4432 and 4406 plants were used for the development of CSSLs in the Koshihikari and Takanari backgrounds, respectively.

Two rice cultivars, Takanari and Koshihikari, developed in Japan (Figure 1), were used to develop the reciprocal CSSLs using the procedure summarized in Figure 2. We conducted repeated reciprocal backcrossing and performed foreground (but not background) selection for the target chromosome segments until the BC_3_F_1_ generation. From the BC_4_F_1_ populations, all heterozygous regions were surveyed, and foreground and background selection were combined to select CSSLs. PCR-based DNA markers (*n* =141), including the previously developed gene markers *GN1a*, *sd1*, and *APO1* [10,16,19,24-26], were used for MAS.

To develop CSSLs in the Koshihikari genetic background, the F_1_ plants derived from a cross between Koshihikari and Takanari were backcrossed to Koshihikari to produce 95 BC_1_F_1_ plants. Then, we used MAS to select 23 BC_1_F_1_ plants carrying one or two target chromosome segments, based on the genotypes of 86 DNA markers distributed across the genome. These 23 BC_1_F_1_ plants were again backcrossed to Koshihikari to produce BC_2_F_1_ seeds. We subsequently grew 408 BC_2_F_1_ individuals derived from the 23 BC_1_F_1_ plants, and selected 24 BC_2_F_1_ plants, carrying one or two heterozygous target segments, by MAS for the subsequent backcross to Koshihikari to produce BC_3_F_1_ seeds. In the same way, 25 out of 518 BC_3_F_1_ individuals derived from the 24 BC_2_F_1_ plants were selected by MAS for subsequent backcross to Koshihikari to produce BC_4_F_1_ seeds. We surveyed the genotypes of the 25 BC_3_F_1_ plants by 141 genome-wide DNA markers for the subsequent target and background selection. Then, 39 out of 509 BC_4_F_1_ individuals derived from the 25 BC_3_F_1_ plants were selected by MAS for all heterozygous regions, including target segments. To obtain candidate plants as CSSLs homozygous for Takanari for the target segments, the 39 BC_4_F_1_ plants were self-pollinated, and the resulting 1606 BC_4_F_2_ individuals were surveyed by MAS to select 41 BC_4_F_2_ plants. Heterozygous segments for the non-target background remained in the 25 BC_4_F_2_ plants, so additional self-pollination and MAS were conducted to minimize the proportion of heterozygous regions in the background. Finally, 41 plants were selected as CSSLs (Figure 2A).

The CSSLs in the Takanari genetic background were developed using the same method as used for the Koshihikari background (Figure 2B). Finally, 39 plants were selected as CSSLs. Seeds of the reciprocal CSSLs can be obtained from the Rice Genome Resource Center (http://www.rgrc.dna.affrc.go.jp/index.html).

### Yield trials

Yield trials were conducted in the experimental paddy field at the NARO Institute of Crop Science, Tsukubamirai (36°02′N, 140°04′E), Ibaraki, Japan, in 2011 and 2012. The soils were Gleyic Fluvisols. Reciprocal CSSLs (41 in the Koshihikari background and 39 in the Takanari background) and parent cultivars (Koshihikari and Takanari) were cultivated under irrigated conditions. Two paddy fields were prepared and each reciprocal CSSL was grown in each paddy field. Seeds were sown in a seedling nursery box on April 26, 2011, and April 25, 2012, and were transplanted (one seedling per hill) on May 19, 2011, and May 17, 2012, respectively. The planting density was 22.2 hills m^−2^, with 15 cm between hills and 30 cm between rows. The experimental plots (5.7 m^2^ each) were arranged in a randomized complete block design with three replications. Basal fertilizer was applied at a rate of 6 g N m^−2^ as controlled release fertilizer (2 g LP40, 2 g LPs100, and 2 g LP140), 5.2 g P m^−2^, and 7.5 g K m^−2^. LP40 and LP140 release 80% of their total nitrogen content at a uniform rate up to 40 and 140 days after application, respectively, at 20–30°C. LPs100 releases 80% of its total nitrogen content at a sigmoid rate up to 100 days after application at 20–30°C.

Days-to-heading was defined as the number of days from sowing to heading of the first panicle in five plants for each CSSL and parent cultivar. At maturity, in mid- to late September, plants covering 1.8 m^2^ (40 hills) were harvested from each plot for determination of yield and its components. Panicle number was counted and the panicles were threshed to obtain unhulled grains, which were weighed and divided equally into subsamples A and B. Approximately 40 g of unhulled grains (subsample C) was selected from subsample A and counted using an electronic seed counter (KC-10S, Fujiwara Scientific Co. Ltd., Tokyo, Japan). Spikelet number per unit area (m^2^) was calculated as the grain number in subsample C divided by the weight of subsample C and multiplied by the total weight of the unhulled grains per unit area. Spikelet number per panicle was calculated as the spikelet number per unit area divided by panicle number per unit area. The hulls from subsample B were subsequently removed with a rice huller (25M, Ohya Tanzo G.K. Company, Aichi, Japan), and the hulled grains were weighed to determine brown rice yield. The grains were then screened using a grain sorter (TWS, Satake Co. Ltd., Tokyo, Japan) with 1.6 mm sieve size and 1000-grain weight was calculated. Ripening percentage was calculated from the number of screened hulled grains divided by the spikelet number per unit area. Brown rice yield and 1000-grain weight were adjusted to 15% moisture content. Culm length was measured for five plants in each CSSL and parent cultivar at maturity.

### Statistical and genetic analyses

Statistical analyses were performed using a general linear model with SPSS 22.0 (IBM, Chicago, IL). CSSL was considered as a fixed effect, and year and replication were considered as random effects. Analysis of variance (ANOVA) was conducted to examine the effects of CSSL on yield and its components. Based on the ANOVA results, significant CSSL effects (*P* <0.05) were explored using Dunnett’s test for yield and its components. In the Dunnett’s test, Koshihikari was used as a control in the Koshihikari genetic background and Takanari was used as a control in the Takanari genetic background. To delineate candidate QTL regions, substitution mapping was conducted by comparing overlapping segments among the CSSLs according to our previous study [17].

## Results

### Genotypes of the reciprocal CSSLs

**Figure 3:**
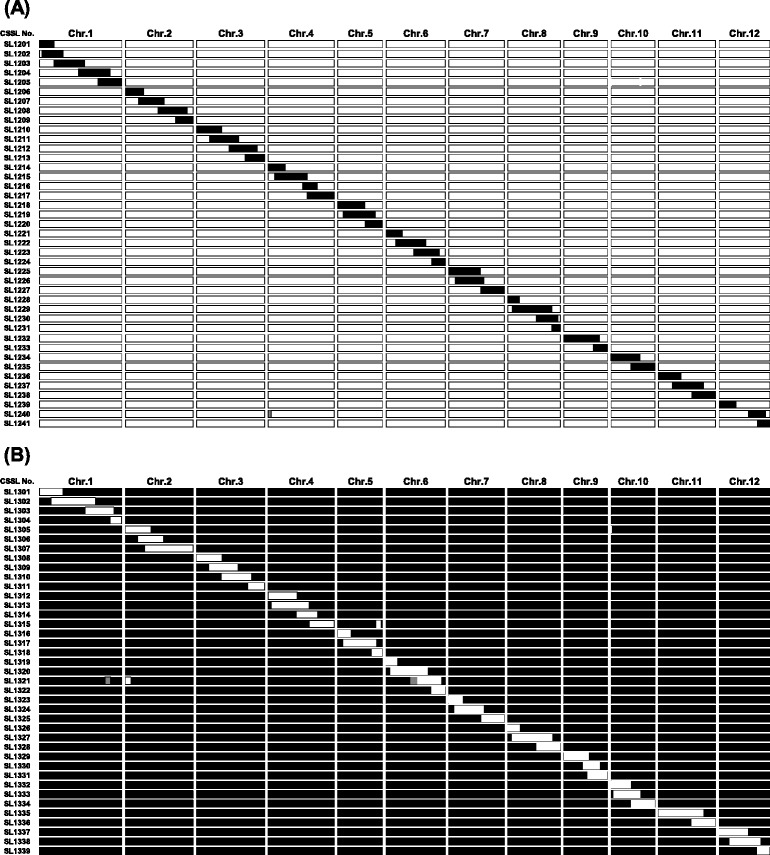
**Graphical genotypes of the reciprocal chromosome segment substitution lines (CSSLs).** We obtained 41 CSSLs in the Koshihikari genetic background **(A)** and 39 CSSLs in the Takanari genetic background **(B)**. White regions denote homozygosity for Koshihikari; black regions denote homozygosity for Takanari; gray regions denote heterozygosity. The graphical genotypes shown here are based on the physical map distance in Os-Nipponbare-Reference-IRGSP-1.0 [27]. Genotype classes of the 141 DNA markers in each CSSL are shown in Additional file 1: Figure S1.

Graphical genotypes of 41 CSSLs in the Koshihikari background and 39 CSSLs in the Takanari background were determined using 141 DNA markers distributed evenly across the 12 rice chromosomes (Figure 3, Additional file 1: Figure S1). Each chromosome was covered by two to five lines carrying overlapping segments, except for a small region between DNA markers RM2935 and RM7344 on chromosome 12 in the Koshihikari background, which was not covered because of sterility when we selected a line carrying the segment homozygous for Takanari. Most CSSLs carried only one chromosome segment. However, a small segment was substituted in the genetic backgrounds in SL1240 and SL1315. SL1321 also carried two heterozygous segments and one homozygous segment for Koshihikari. The substituted segment size in each CSSL ranged from 6.9 Mb to 26.2 Mb in the Koshihikari background and from 7.4 Mb to 27.1 Mb in the Takanari background.

### Climate conditions and variation in days-to-heading in the reciprocal CSSLs

**Figure 4:**
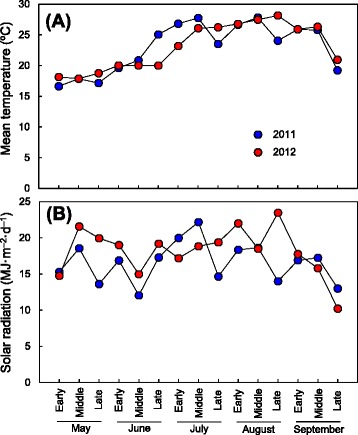
**Mean temperature and solar radiation.** Mean temperature **(A)** and solar radiation **(B)** measured at the experimental paddy field were calculated as the average values from the beginning, middle, and end of each month.

Mean temperatures during the experimental period were similar in 2011 and 2012, and showed a gradual increase as the season progressed, until mid-September (Figure 4A). Solar radiation was relatively higher in late May and late August in 2012 compared with 2011 (Figure 4B). Koshihikari headed at approximately 99 days after sowing, and Takanari headed at 102–103 days after sowing (Additional file 2: Figure S2). In the Koshihikari background, SL1222 and SL1208 headed approximately 11 days earlier and 11 days later than Koshihikari, respectively. Earlier heading in SL1222 may be derived from the effect of *Hd1*, because the substituted segment in SL1222 contained *Hd1* [28]. Late heading in SL1208 is discussed later. In the remaining 39 CSSLs, days-to-heading ranged from 95 to 105 (within 6 days of Koshihikari). In the Takanari background, SL1320 and SL1323 did not head under the experimental conditions. No heading in SL1320 may be caused by the effect of *Hd1*, because the substituted segment in SL1320 contained *Hd1* [28]. No heading in SL1323 may be caused by a new QTL because no QTL has been reported in the substituted region on the short arm of chromosome 7. In addition, SL1335 and SL1336 headed 17 and 29 days later than Takanari, respectively. Late heading in SL1335 and SL1336 is discussed later. On the other hand, the remaining 35 CSSLs headed at 97–108 days after sowing, which was also within 6 days of Takanari. Therefore, we considered that most CSSLs and parent cultivars were grown under similar climate conditions.

### Yield and its components in the reciprocal CSSLs

**Figure 5:**
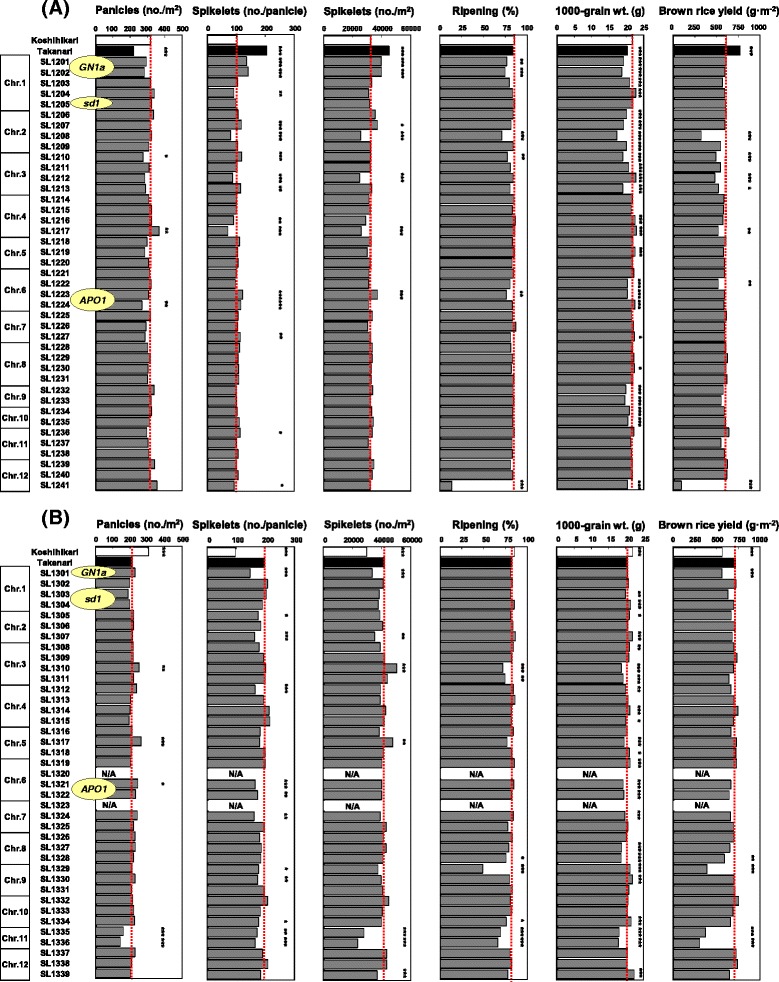
**Yield and its components for the chromosome segment substitution lines (CSSLs) in the Koshihikari (A) and Takanari (B) backgrounds.** Bars indicate mean values over two years. Dashed red lines denote trait values in Koshihikari **(A)** and Takanari **(B)**. ***P <0.001, **P <0.01 and *P <0.05 versus Koshihikari **(A)** and Takanari **(B)**, assessed by Dunnett’s test. N/A, not available. *GN1a*, *sd1*, and *APO1* adjacent to the name of a CSSL indicate that the CSSL carries that gene.

Takanari produced approximately 40% more spikelets per unit area than Koshihikari, which was a result of Takanari having 30% fewer panicles but twice as many spikelets per panicle (Figure 5). The same ripening percentage was obtained in Takanari and Koshihikari, although 1000-grain weight was 7% lower in Takanari. Finally, brown rice yield was 27% higher in Takanari than in Koshihikari.

Previous studies identified and cloned QTLs for sink-size traits (spikelet number per panicle), including *GN1a* [29] and *APO1* [30], and a semidwarf gene, *sd1* [31], as a single gene. Because we found sequence differences between Takanari and Koshihikari in these genes (Additional file 3: Figure S3), we first focused on the effects of the genes. SL1201 and SL1202, which carried the Takanari allele of *GN1a* in the Koshihikari background, produced 33% and 38% more spikelets per panicle and 22% and 23% more spikelets per square meter than Koshihikari, respectively (Figure 5A). However, these two CSSLs reduced ripening percentage and 1000-grain weight, and there was no difference in final brown rice yield between these CSSLs and Koshihikari. Meanwhile, SL1301, carrying the Koshihikari allele of *GN1a* in the Takanari background, produced 26% fewer spikelets per panicle and 20% fewer spikelets per square meter than Takanari (Figure 5B). Ripening percentage and 1000-grain weight in SL1301 were the same as in Takanari, and the final brown rice yield in SL1301 was 22% lower than that in Takanari. Similar reciprocal effects of *APO1* were observed for spikelet number per panicle; SL1223 and SL1224, which contained the Takanari allele of *APO1*, produced 19% and 13% more spikelets per panicle, respectively, whereas SL1321 and SL1322 (containing the Koshihikari allele of *APO1*) produced 17% and 12% fewer spikelets per panicle, respectively. However, the effects of *APO1* did not lead to changes in final brown rice yield. Reciprocal effects were confirmed for culm length on the *sd1* gene; SL1205 (carrying the Takanari allele of *sd1*) had shortened culms compared with Koshihikari, whereas SL1303 and SL1304 (carrying the Koshihikari allele of *sd1*) had elongated culms compared with Takanari (Additional file 2: Figure S2). However, no effects of *sd1* were observed for brown rice yield and its components in the reciprocal backgrounds.

In addition to the CSSLs carrying *GN1a*, *APO1*, and *sd1*, there were significant differences in brown rice yield and its components between Koshihikari and some CSSLs in the Koshihikari genetic background (Figure 5A), and between Takanari and some CSSLs in the Takanari genetic background (Figure 5B). Although some CSSLs had positive values for a yield component, they did not produce significantly higher yield than the recurrent parental cultivar in both backgrounds. For example, SL1310 (Takanari background) produced 19% more panicles and 20% more spikelets per square meter than Takanari, but had reduced ripening percentage and 1000-grain weight. Therefore, the final brown rice yield in this CSSL was similar to that in Takanari.

### QTL mapping for yield and its components

**Figure 6:**
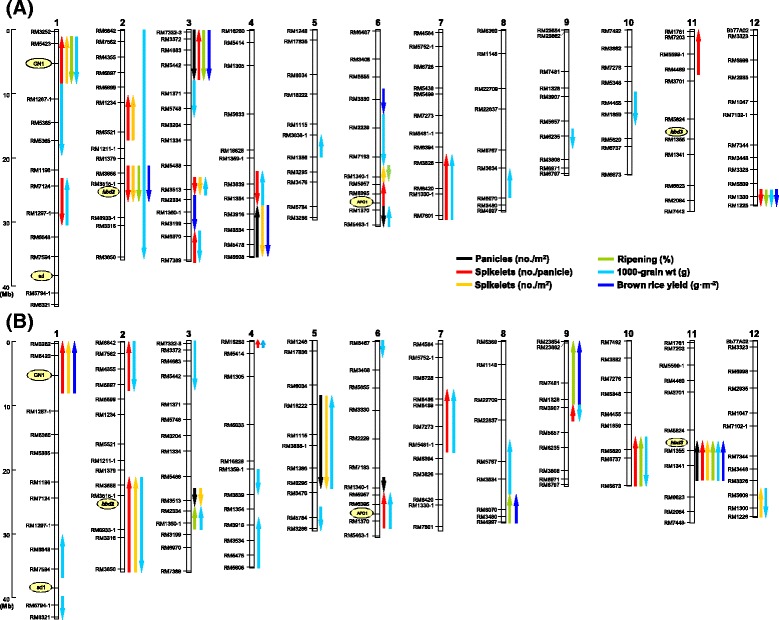
**Substitution mapping of quantitative trait loci (QTLs) for yield and its components by comparing overlapping segments among chromosome segment substitution lines (CSSLs).** QTLs in the Koshihikari **(A)** and Takanari **(B)** backgrounds. Chromosome numbers are indicated above each physical map. Marker names are located to the left of each chromosome. Colored arrows denote putative QTLs for yield and its components. Upward and downward arrowheads indicate that the trait value was increased by the Takanari or Koshihikari allele, respectively.

We detected 48 and 47 QTLs for yield and its components in the Koshihikari and Takanari backgrounds, respectively (Figure 6).

In the Koshihikari background, three QTLs for panicle number were identified, one of which increased and two of which decreased panicle number in plants with the Takanari allele (Figure 6A). Twelve QTLs for number of spikelets per panicle were detected, seven with positive effects and five with negative effects on spikelet number in plants with the Takanari allele. Considering the effects of the QTLs and the chromosomal regions, the loci on the short arm of chromosome 1 and on the long arm of chromosome 6 were regarded as *GN1a* and *APO1*, respectively. Six QTLs were found for spikelet number per square meter; half of these increased and half decreased the spikelet number in plants with the Takanari allele. The five QTLs identified for ripening percentage all had negative effects on plants with the Takanari allele. Sixteen QTLs were detected for 1000-grain weight; of these, seven increased and nine reduced the value of this yield component in plants with the Takanari allele. Six QTLs were identified for brown rice yield, and all had negative effects on plants with the Takanari allele (Figure 6A).

In the Takanari background, four QTLs were found for panicle number, three of which increased and one of which decreased panicle number in plants with the Koshihikari allele (Figure 6B). Nine QTLs for number of spikelets per panicle were detected, and all had negative effects in plants with the Koshihikari allele. Considering the effects of the QTLs and the chromosomal regions, the loci on the short arm of chromosome 1 and on the long arm of chromosome 6 were regarded as *GN1a* and *APO1*, respectively. Six QTLs were found for number of spikelets per square meter; two increased and four decreased spikelet number in plants with the Koshihikari allele. Five QTLs for ripening percentage had negative effects in plants with the Koshihikari allele. Nineteen QTLs were detected for 1000-grain weight; ten increased and nine decreased 1000-grain weight in plants with the Koshihikari allele. Four QTLs for brown rice yield were identified; all had negative effects on yield in plants with the Koshihikari allele.

## Discussion

Rice yield is a highly complex trait and is controlled by a large number of QTLs with small individual effects. CSSLs are appropriate for detecting QTLs with both large and small effects, and reciprocal CSSLs confer the advantage of enabling evaluation of differences in allelic effects of QTLs in both genetic backgrounds. If a detected QTL shows the same gene activity in reciprocal genetic backgrounds, that locus should have no genetic interaction or epistasis with other background factor(s). QTLs that show different gene activity in the reciprocal backgrounds may be involved in genetic interaction or epistasis with other background factor(s) [21]. By using reciprocal CSSLs derived from a cross between Takanari and Koshihikari, we detected a number of QTLs underlying brown rice yield and its components. Among these loci, we confirmed that the Takanari alleles of *GN1a* and *APO1* increased the number of spikelets per panicle in the reciprocal backgrounds (Figures 5 and 6). A QTL for the number of spikelets per unit area, at RM3513 on chromosome 3, and QTLs for 1000-grain weight at RM3634 and RM1300 on chromosomes 8 and 12, respectively, exhibited the same gene activity in both genetic backgrounds. These results indicate that these five QTLs should be a single factor, unless multiple genes associated with the trait were located in the substituted segment. Thus, favorable alleles from these QTLs can be used to improve target traits in either of the reciprocal genetic backgrounds. Furthermore, a recent study cloned a QTL on the long arm of chromosome 8 as *GW8* controlling grain size [32]. Because the position of the QTL for 1000-grain weight is close to *GW8*, there is a possibility that the QTL detected in this study is *GW8*. Further study is necessary to confirm this.

On the other hand, the remaining QTLs were detected in only one of the two genetic backgrounds, suggesting that these loci may be under epistatic control with other gene(s) in the background. A possible epistatic interaction was observed for QTL clusters at RM3515-1 on chromosome 2 in the Koshihikari background and at RM1355 on chromosome 11 in the Takanari background (Figure 6). These QTL clusters were not detected at the same genomic regions in the opposite background. SL1208, carrying the QTL cluster on chromosome 2, and SL1335 and SL1336, which carried the QTL cluster on chromosome 11, showed hybrid weakness (delayed heading, dwarf plant stature, fewer spikelets, lower ripening percentage, and lower yield) (Figure 5, Additional file 2: Figure S2). A previous study revealed that hybrid breakdown is caused by interaction of two recessive genes, *hbd2* and *hbd3*, and that Koshihikari carries *hbd3* and an *indica* cultivar, Habataki, which is a sister line to Takanari, carries *hbd2* [33]. The *hbd2* and *hbd3* genes are located in the vicinity of QTL clusters on chromosomes 2 and 11, respectively. Assuming that Takanari also carried *hbd2*, the two QTL clusters and hybrid weakness observed in SL1208, SL1335, and SL1336 can be well explained because SL1208 should have carried *hbd2* and *hbd3* in the Koshihikari background and SL1335 and SL1336 also carried *hbd2* and *hbd3* in the Takanari background. Therefore, the two QTL clusters should be a result of interaction between *hbd2* and *hbd3*. Although we could not elucidate gene interactions for other QTLs detected in only one of the reciprocal backgrounds, detection of many QTLs in only one genetic background suggests that a large part of the variation in yield and its components between Takanari and Koshihikari may be controlled by gene interactions. This is the first study to suggest the possibility of epistatic control of yield and its components on the basis of reciprocal CSSLs. Further studies are necessary to identify background factors that interact with the detected QTLs.

Mapping of QTLs also revealed a trade-off among yield components. A notable example was that the allele of the QTL associated with increased sink size was associated with decreased ripening percentage or 1000-grain weight. This trade-off was observed for seven chromosomal regions in the Koshihikari background (including *GN1a* and *APO1*) and six in the Takanari background (Figure 6). The trade-off might be caused by a shortage of source strength or carbohydrate translocation, or might be caused by an imbalance among sink size, source strength, and translocation capacity, resulting in no increase in final yield. A lack of remarkable increase in grain yield was also reported for NILs containing the favorable allele of *GN1a* and *APO1* in other *japonica* genetic backgrounds [34]. However, it should be noted that the parental cultivar, Takanari, obtained a high ripening percentage despite a large sink size derived from the favorable allele of *GN1a* and *APO1*. The high ripening percentage in Takanari is considered to be caused by the strong source (high photosynthesis rate) and high carbohydrate translocation capacity [11-14]. These results suggest that QTLs for increased source strength and translocation capacity should be found in the Takanari allele and that the combination of *GN1a* or *APO1* with these QTLs would be necessary to attain higher yield in the Koshihikari background. Recently, a QTL for high leaf photosynthesis was identified in Takanari and cloned as a single gene (*GPS*) in the same genetic combination between Takanari and Koshihikari [35]. We are currently developing a pyramid line carrying *GN1a* and *GPS* to test yield increase in the Koshihikari background.

Although the Takanari allele of *GN1a* did not contribute to yield increase in the Koshihikari background, the Koshihikari allele of *GN1a* decreased sink size traits (spikelet number per panicle and per square meter) and thus reduced brown rice yield in the Takanari background (Figure 5B and Figure 6B). These results indicate that the Takanari allele of *GN1a* is required to achieve high yield potential in Takanari. These results and the strong source in Takanari also imply that yield potential in Takanari might be increased by enlarging its sink size. We identified a QTL that increased panicle number and spikelet number per square meter on the long arm of chromosome 3 in the Takanari background (Figure 6B). SL1310, which carried this QTL, produced 20% more spikelets per square meter than Takanari. This is a promising QTL to increase sink size in Takanari. However, SL1310 did not attain higher brown rice yield than Takanari, because of reduced ripening percentage and 1000-grain weight (Figure 5B). These results indicate that it is necessary, even in the Takanari background, to combine the QTL on chromosome 3 with loci that enhance source strength and translocation capacity to raise yield potential. Although Koshihikari has lower leaf photosynthesis than Takanari, our previous study detected a QTL for increasing leaf photosynthesis with the Koshihikari allele in the Takanari background [35]. We are currently combining the QTL on chromosome 3 with the high-photosynthesis QTL in the Takanari background to test the increase in yield potential in Takanari, as well as attempting to clone both QTLs.

We detected six QTLs for yield in the Koshihikari background and four in the Takanari background, but no QTLs for yield common to both backgrounds were detected, and all QTLs had negative effects on yield. As discussed above, the failure to detect common QTLs might be caused by epistatic control of these QTLs. The negative effects might be due to imbalance among sink size, source strength, and translocation capacity caused by the substitution of a QTL. However, the results presented here are based on trials conducted at a single experimental site with a single fertilization treatment. Because yield is often influenced by environmental conditions, further trials under multiple environmental conditions are necessary to confirm the effects of the QTLs detected in this study.

## Conclusion

We have successfully developed reciprocal CSSLs derived from a cross between rice cultivars Takanari and Koshihikari. Genetic analysis by reciprocal CSSLs confirmed their usefulness and indicated that some QTLs for yield and its components represented a single factor, while others may be controlled by epistatic interactions. Substitution mapping also suggested the need to combine sink-size QTLs with source-strength or translocation-capacity QTLs to increase rice yield in both genetic backgrounds. Our results provide a foundation for developing rice cultivars with higher yield potential in future breeding programs.

## Additional files

Additional file 1: Figure S1. Genotype data for 41 chromosome segment substitution lines (CSSLs) in the Koshihikari genetic background and 39 CSSLs in the Takanari genetic background. Columns show CSSLs and rows show DNA markers. A, B, and H indicate homozygous for Koshihikari, homozygous for Takanari, and heterozygous, respectively. Approximate positions (Mb) of each marker are based on the physical map distance in Os-Nipponbare-Reference-IRGSP-1.0 [27]. The sequences of the forward and reverse primers were 5′-CTCCATCCCAAAATAAGTTC-3′ and 5′-GCTGGCCTGTCATCC-3′ for *GN1a*, 5′-AGCTGGACATGCCCGTGGTC-3′ and 5′-TTGAGCTGCTGTCCGCGAAG-3′ for *sd1*, and 5′-CCGGTTTTGGTTTGTCTCAG-3′ and 5′-ATGAACACTGTCCAACAAATTGTTT-3′ for *APO1*, respectively. Additional file 2: Figure S2. Days-to-heading and culm length of chromosome segment substitution lines (CSSLs) in the Koshihikari (A) and Takanari (B) backgrounds. Bars indicate mean values over two years. Dashed red lines denote trait values in Koshihikari (A) and Takanari (B). ****P* <0.001, ***P* <0.01, and **P* <0.05 versus Koshihikari (A) and Takanari (B), determined by Dunnett’s test. N/A, not available. Additional file 3: Figure S3. Sequence polymorphisms of *GN1a*, *APO1*, and *sd1* between Koshihikari and Takanari. Light blue bars represent exons; white bars represent 5′ and 3′ untranslated regions.
