# Supplementary figures and images for: Genetic mechanisms underlying yield potential in the rice high-yielding cultivar Takanari, based on reciprocal chromosome segment substitution lines
Source: BMC Plant Biol. 2014 Nov 18;14:295. doi: 10.1186/s12870-014-0295-2 (PMC4243286; doi:10.1186/s12870-014-0295-2)

**(A)**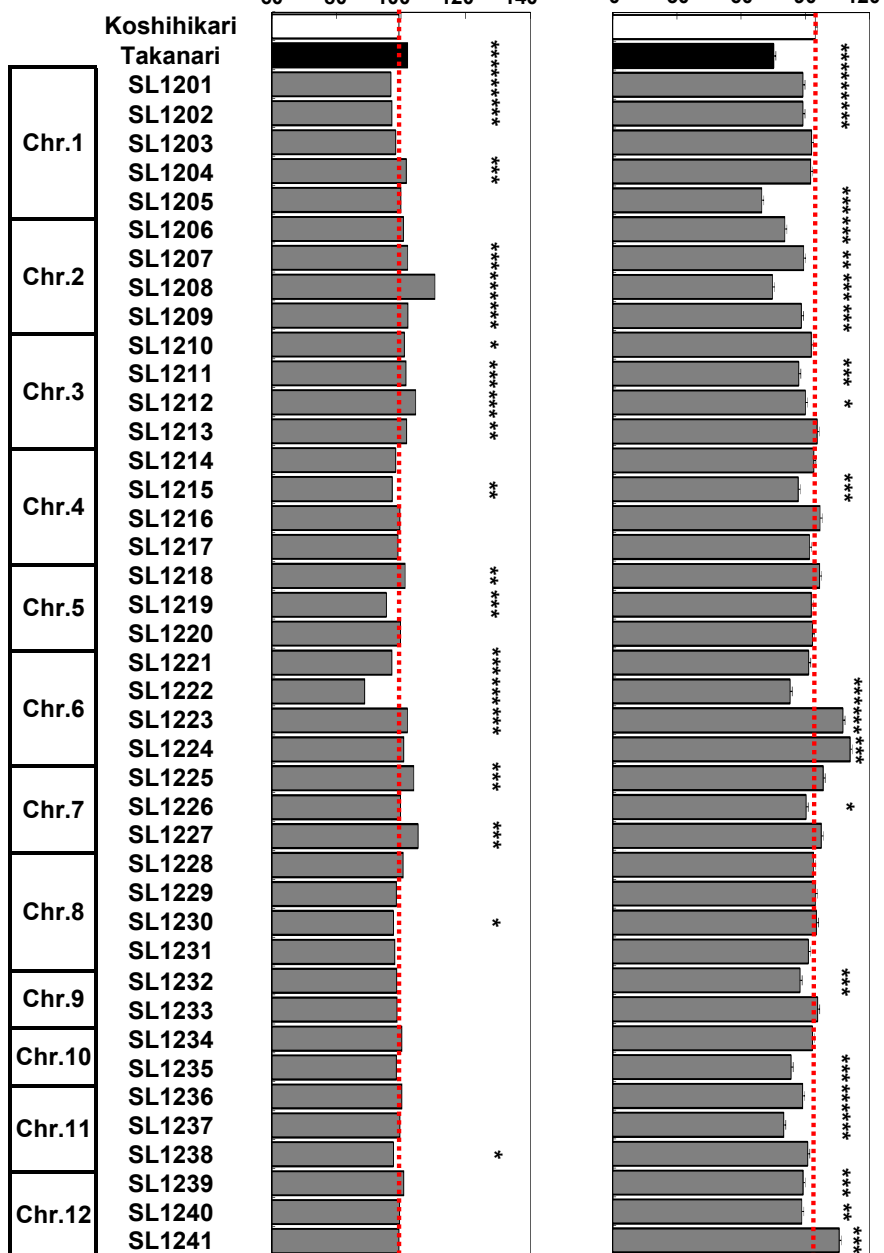**(B)**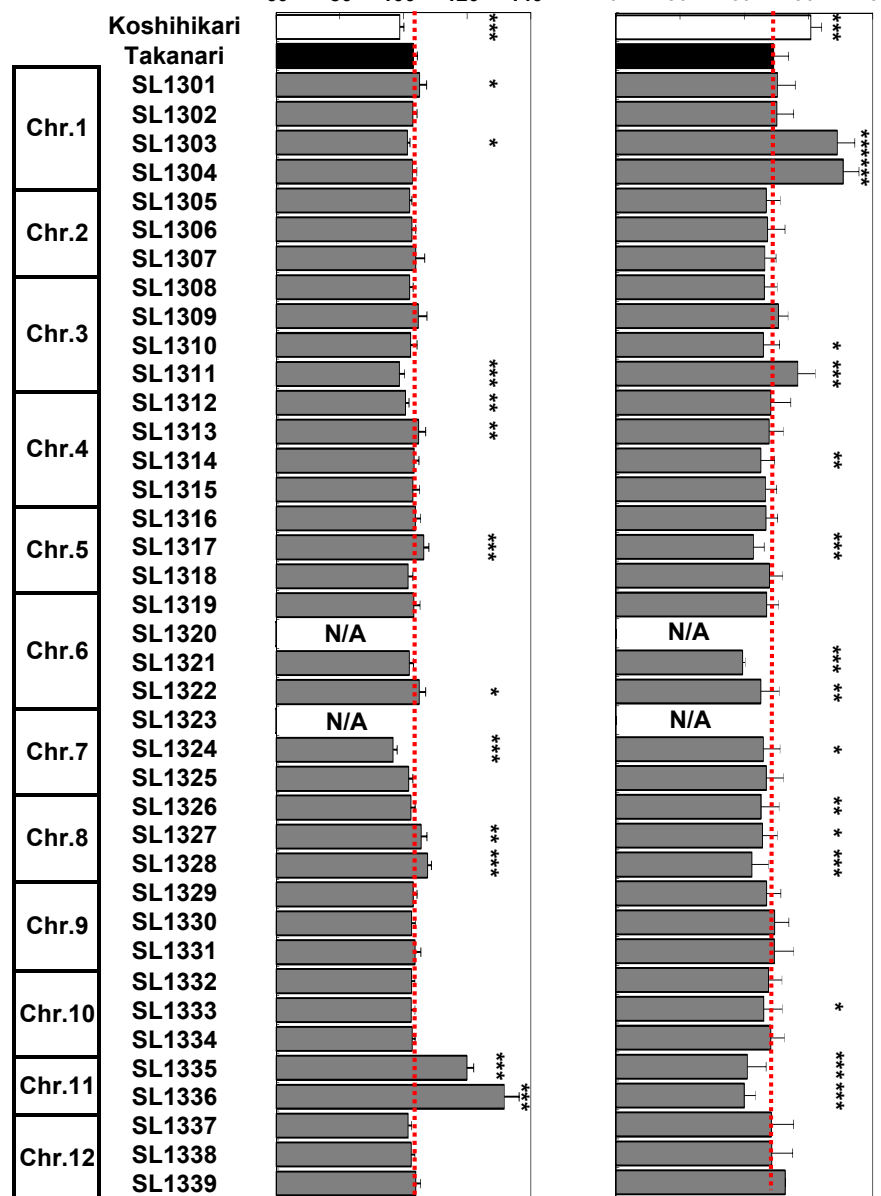

Supplement: Additional file 2: Figure S2. — Days-to-heading and culm length of chromosome segment substitution lines (CSSLs) in the Koshihikari (A) and Takanari (B) backgrounds. Bars indicate mean values over two years. Dashed red lines denote trait values in Koshihikari (A) and Takanari (B). ***P <0.001, **P <0.01, and *P <0.05 versus Koshihikari (A) and Takanari (B), determined by Dunnett’s test. N/A, not available. [file 12870_2014_295_MOESM2_ESM.pdf]

## GN1a

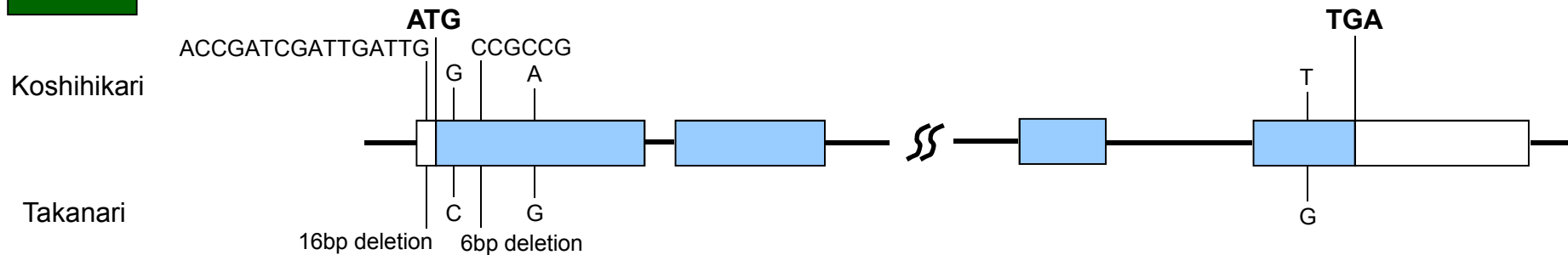

## APO1

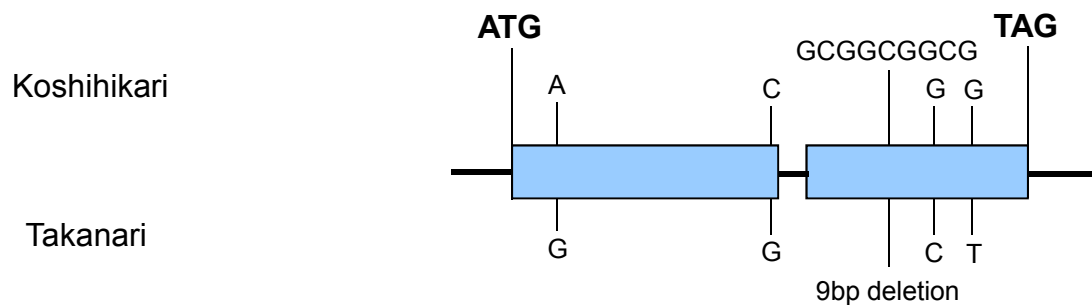

## sd1

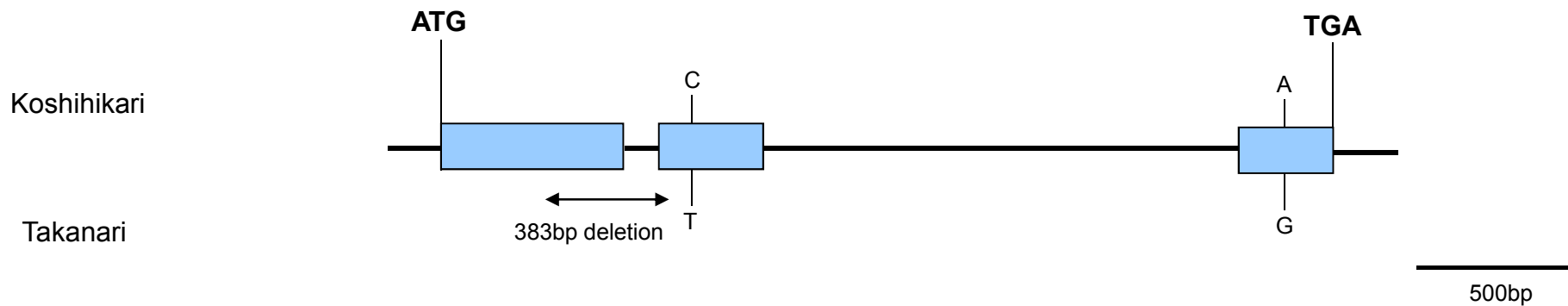

Supplement: Additional file 3: Figure S3. — Sequence polymorphisms of GN1a, APO1, and sd1 between Koshihikari and Takanari. Light blue bars represent exons; white bars represent 5′ and 3′ untranslated regions. [file 12870_2014_295_MOESM3_ESM.pdf]
